# Dorsal Striatum Transcriptome Profile Profound Shift in Repeated Aggression Mouse Model Converged to Networks of 12 Transcription Factors after Fighting Deprivation

**DOI:** 10.3390/genes13010021

**Published:** 2021-12-22

**Authors:** Vladimir Babenko, Olga Redina, Dmitry Smagin, Irina Kovalenko, Anna Galyamina, Roman Babenko, Natalia Kudryavtseva

**Affiliations:** 1 FRC Institute of Cytology and Genetics, Siberian Branch of Russian Academy of Sciences, 630090 Novosibirsk, Russia; oredina@bionet.nsc.ru (O.R.); smagin@bionet.nsc.ru (D.S.); koir@bionet.nsc.ru (I.K.); galyamina@bionet.nsc.ru (A.G.); babe-roman@yandex.ru (R.B.); natnik@bionet.nsc.ru (N.K.); 2 Pavlov Institute of Physiology, Russian Academy of Sciences, 199034 Saint Petersburg, Russia

**Keywords:** dorsal striatum, nucleus accumbens, transcriptome, cAMP cascade, dopamine, addiction, endogenous opioids, behavior, chronic social conflict

## Abstract

Both aggressive and aggression-deprived (AD) species represent pathologic cases intensely addressed in psychiatry and substance abuse disciplines. Previously, we reported that AD mice displayed a higher aggressive behavior score than the aggressive group, implying the manifestation of a withdrawal effect. We employed an animal model of chronic social conflicts, curated in our lab for more than 30 years. In the study, we pursued the task of evaluating key events in the dorsal striatum transcriptome of aggression experienced mice and AD species compared to controls using RNA-Seq profiling. Aggressive species were subjected to repeated social conflict encounters (fights) with regular positive (winners) experience in the course of 20 consecutive days (A20 group). This led to a profoundly shifted transcriptome expression profile relative to the control group, outlined by more than 1000 differentially expressed genes (DEGs). RNA-Seq cluster analysis revealed that elevated cyclic AMP (cAMP) signaling cascade and associated genes comprising 170 differentially expressed genes (DEGs) in aggressive (A20) species were accompanied by a downturn in the majority of other metabolic/signaling gene networks (839 DEGs) via the activation of transcriptional repressor DEGs. Fourteen days of a consecutive fighting deprivation period (AD group) featured the basic restoration of the normal (control) transcriptome expression profile yielding only 62 DEGs against the control. Notably, we observed a network of 12 coordinated DEG Transcription Factor (TF) activators from 62 DEGs in total that were distinctly altered in AD compared to control group, underlining the distinct transcription programs featuring AD group, partly retained from the aggressive encounters and not restored to normal in 14 days. We found circadian clock TFs among them, reported previously as a withdrawal effect factor. We conclude that the aggressive phenotype selection with positive reward effect (winning) manifests an addiction model featuring a distinct opioid-related withdrawal effect in AD group. Along with reporting profound transcriptome alteration in A20 group and gaining some insight on its specifics, we outline specific TF activator gene networks associated with transcriptional repression in affected species compared to controls, outlining *Nr1d1* as a primary candidate, thus offering putative therapeutic targets in opioid-induced withdrawal treatment.

## 1. Introduction

An aggression-tackling program manifests one of the key issues in health services due to the prevalence of aggression in societies across the world [1]. Consequently, studies on the neurologic mechanisms of aggression attract major psychiatric attention, along with depression stated. Notably, chronic aggression may manifest as an addiction-like state [2,3,4].

Currently, it is established that the dorsal striatum (DS) and nucleus accumbens (NAcc) in the ventral part of the striatum manifest the coordinated regulation of motor activity and stereotypical behaviors [5]. They are reported to be involved in a variety of cognitive, reward and social hierarchy maintenance and learning processes [6]. In particular, the dorsal striatum has been proven to supervise the consolidation of the new response upon the learning process [6]. Hence, these brain regions are inherently involved in addictive, depressive and aggressive behavior [7,8,9] when regularly exposed to a stressful environment or substance abuse, thus leading to behavior pattern fixation. 

The major neuron body of both brain regions (DS, NAcc) comprises GABAergic medium spiny neurons (MSN), acting quite synchronously upon the response to phasic dopamine firing [10]. In particular, using RNA-Seq transcriptome data sampled from the DS of mice in a chronic social conflict experiment, we previously reported distinct coordinately expressed gene cluster profiles corresponding to D1- and D2- MSNs in certain phases [11]. The major mechanism of MSN intracellular signal processing upon dopamine/glutamate monoamine uptake is cAMP-mediated (de)phosphorylation signaling cascades, with multiple phosphoproteins involved [7,8,12].

Notably, it is reported that dopamine metabolism correlates with endogenous opioid synthesis in the striatum [13,14], implying the opioid-mediated alteration of synaptic plasticity in affected MSNs. Multiple reports on opioid-mediated changes report particular alterations in the structural and functional plasticity of dendritic spines on MSNs [15,16]. The morphological dendritic changes invoked by opioids are exemplified by the actin cytoskeleton’s remodeling [17]. The actin cytoskeleton gene expression alteration is subject to membrane dynamics such as cell motility and morphogenesis. In particular, dendritic spine dynamics (formation and elimination) are supervised by the actin cytoskeleton.

Genes encoding for cytoskeleton regulatory proteins are affected by opioids. The GTPases family, involved in regulating the actin cytoskeleton, is attenuated by opioid administration [18]. Transcription factors such as *AP-1/Fos* account for the regulation of approximately a quarter of the structural and synaptic genes, such as activity-regulated cytoskeletal proteins and others [19]. Besides neurons themselves, astrocytes also take part specifically in pruning synapses [20]. Thus, a great deal of changes upon opioid administration take place specifically at the synaptic structural level in MSNs [21].

In each experiment, we tracked the behavior of all males, recording videos of their behavior during agonistic interactions, which allowed us to identify the most aggressive mice demonstrating the greatest daily number and duration of attacks, hyperactivity, the number of behavioral stereotypes, etc. Those with the most eminent aggressive phenotypes (long-lasting, pronounced aggression toward any losers each day) were selected for transcriptome analysis. Winners demonstrated an increased aggression score after a 14-day period of deprivation (AD group) in comparison with the aggression level before deprivation [2,3].

Notably, chronic aggression accompanied by a positive reward (win) inherently manifests an addiction state upon positive fighting experience rounds in mice [2,3,4]. Namely, signs of addiction behavior, similar to drug users, according to Robinson and Berridge, 2003 [22], were observed in mice with repeated experience of aggression supported by wins in daily agonistic interactions [4,23]. In particular, we observed activation of the brain’s dopaminergic and opioidergic systems and the development of tolerance or sensitization to dopaminergic and opiodergic receptor antagonists or agonists after chronic aggression experience [4]. Similar to the withdrawal effect among substance abusers, the experienced winners demonstrated increased aggression after a period of fighting deprivation [3].

Using a chronic social conflict model of aggressive and aggression-deprived nurtured mice, this study pursues the aim of elucidating the basic differentially expressed gene network dynamics characterizing both affected groups.

## 2. Materials and Methods

### 2.1. Animals

Adult male C57BL/6 mice were obtained from the Animal Breeding Facility of the FRC Institute of Cytology and Genetics SB RAS (Novosibirsk, Russia). Animals were housed under standard conditions (12:12 hr light/dark regime starting at 8:00 am, with food in pellets and water available ad libitum). Mice were weaned at three weeks of age and housed in groups of 8–10 in standard plastic cages (36 × 23 × 12 cm). Experiments were performed with 10–12-week-old animals. All procedures were in compliance with the European Communities Council Directive 210/63/EU on 22 September 2010. The study was approved by Scientific Council N 9 of the Institute of Cytology and Genetics SB RAS of 24 March 2010, N 613 (Novosibirsk). 

### 2.2. Experimental Procedures

#### Modeling Repeated Aggression in Male Mice 

Repeated negative and positive social experiences in male mice were induced by daily agonistic interactions with the use of a sensory contact model, which later was renamed the “model of chronic social conflicts” [23,24]. Pairs of male mice were each placed in a cage (28 × 14 × 10 cm) bisected by a perforated transparent partition allowing the animals to hear, see and smell each other, but preventing physical contact. The animals were left undisturbed for two days to adapt to the new housing conditions and for sensory acquaintance before they were exposed to agonistic interactions. Every afternoon (2:00–5:00 p.m. local time), the cage cover was replaced with a transparent one, and 5 min later (the time it took for mice to start reacting to a partner in a neighboring compartment), the partition was removed for 10 min to encourage agonistic interactions. The superiority of one of the mice was firmly established within two or three confrontations with the same opponent. The superior mouse (winner) would be attacking, chasing and biting another, who would be displaying only defensive behavior (withdrawal, sideways postures, upright postures, freezing or lying on the back). As a rule, aggressive interactions between males were discontinued by lowering the partition if the strong attacks lasted for 3 min (in some cases less) to prevent injury to the defeated mice. Each defeated mouse (loser) was exposed to the same winner for three days, while, afterwards, each loser was placed, once a day after the agonistic interactions, in an unfamiliar cage with a previously unencountered winner behind the partition. Each winning mouse (aggressive mouse, winner) remained in its original cage. This procedure was performed for 20 days (once a day) and yielded an equal number of losers and winners.

**Figure 1:**
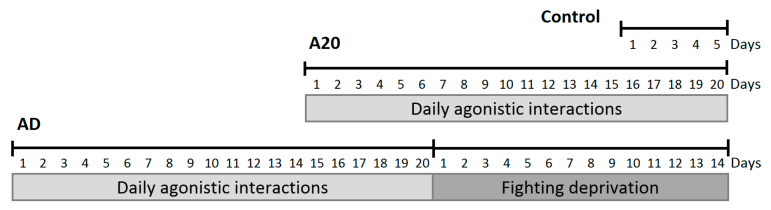
Timescale of experiments. A20—mice with 20 days of consecutive wins in daily agonistic interactions; AD—A20 mice after subsequent 14 days of fight deprivation; Control—mice without agonistic interactions.

Three groups of animals (*n* = 6) were collected in this experiment: (1) controls—mice without experience of agonistic interactions; (2) winners—groups of mice that were repeatedly aggressive for 20 days (A20); (3) aggression-deprived mice (AD) that were converted from winners after a period of fighting deprivation for 14 days in secluded cages (Figure 1). The winners 24 h after the last agonistic interaction, the control animals and AD were decapitated simultaneously. The dorsal striatum regions were dissected by the same experimenter according to the map in the Allen Mouse Brain Atlas [25]. All samples were deposited in *RNAlater* solution (Life Technologies, Carlsbad, CA, USA) and were stored at −70 °C prior to the sequencing routine. 

### 2.3. RNA-Seq Data Collection and Processing

The collected brain samples were delivered to JSC Genoanalytica (www.genoanalytica.ru, accessed on 18 December 2021, Moscow, Russia) for RNA-Seq sequencing. mRNA was extracted using a Dynabeads mRNA Purification Kit (Ambion, Thermo Fisher Scientific, Waltham, MA, USA). cDNA libraries were created using the NEBNext mRNA Library PrepReagent Set for Illumina (New England Biolabs, Ipswich, MA USA) according to the manufacturer’s protocol. The Illumina HiSeq 2500 System was used for sequencing using single (non-paired end) reads of 50 bp length. The target coverage was set to 20 Mio. reads per sample.

The dorsal striatum regions were processed for each of 6 animals per group, separately, without technical replicates. Three groups of animals were employed in the study.

The raw reads from RNA-Seq experiments were trimmed for quality (phred ≥ 20) and length (bp ≥ 32) using Trimmomatic v. 3.2.2 [26]. Illumina adapters were trimmed. The reads were then aligned against the GRCm38.p3 reference genome using the STAR aligner [27]. The descriptive statistics of sample mapping are available in Table S1.

The Cuffnorm app of *Cufflinks* suite [28] was employed for expression rate assessment in FPKM units. The *Cuffdiff* app from the same suite was used for elucidating differentially expressed genes.

### 2.4. Statistical Analysis

Principal component analysis (PCA) was employed using the *XLStat* statistical package (www.xlstat.com; accessed on 18 December 2021). Pearson product moment correlation matrix for gene expression in samples was used as input data for PCA. An agglomerative hierarchical clustering (AHC) routine was performed using the Pearson correlation matrix with the same *XLStat* package.

For the analysis, we used DEGs given significant FDR < 0.05 with *cuffdiff* software. We then employed the strategy of DEG analysis and annotation with the string-db.org suite, selecting the following two key thresholds. (a) DEGs should be connected/associated based on evidence provided by string-db. This guarantees the avoidance of random spurious single DEG signals, since a coordinated functional shift is exponentially more statistically robust. (b) The auxiliary evidence of the connected gene neighborhood should be also supported by transcription co-variation in our data, corroborative to external evidence provided by (a). We thus confined our attention/conclusions based primarily on the connected gene neighborhood making most sense in the DEG functional elaboration assisted by Gene Ontology (GO) annotation. If there were unconnected but evidently important DEGs (such as the key transcription factor activators), we used the procedure of string-db-mediated expansion of the corresponding DEG gene neighborhood, with subsequent checking of consistent co-variation of the inferred network in our data.

## 3. Results

### 3.1. Detecting DEGs in Three Pairwise Comparisons

We performed three-way comparisons of control, A20 and AD expression data by means of *CuffDiff* software [28]. The results yielded approximately 1000 DEGs for control vs. A20 species, and only 62 for AD compared with controls (Table 1). This implies that the dorsal striatum state of AD species is quite close to the control, while, in aggressive A20 species, there are a great deal of alterations.

### 3.2. C_AD Comparison

We performed GO annotation of DEGs from C_AD comparison, which yielded a single highly significant GO term annotation (process) “DNA-binding transcription activator activity, RNA polymerase II-specific” (GO:0001228; 12 genes, FDR < 2.6 × 10^−5^).

**Figure 2:**
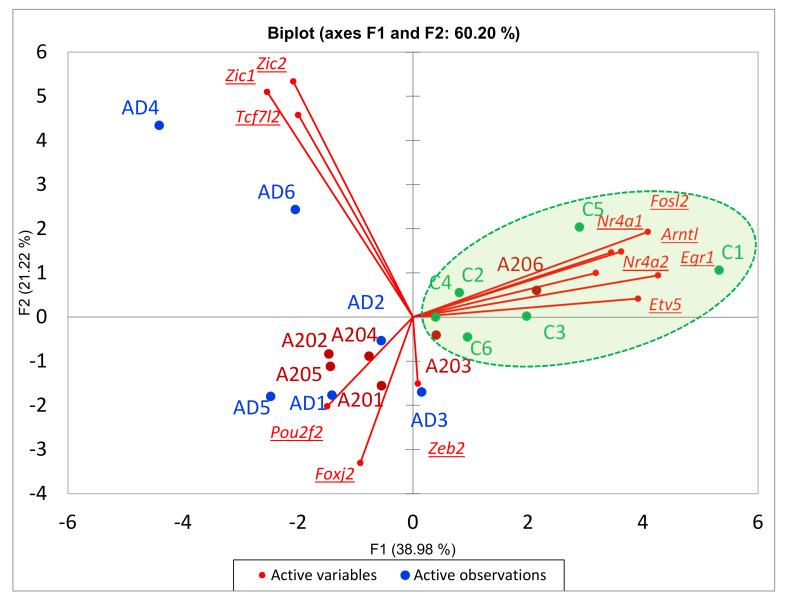
PCA biplot outlines distinct alteration of TF programs (3 DEG clusters) between AD (blue), A20 (red) and control (green) groups. Green shaded area is control group.

To ascertain the 12 DEG set clustering mode outlined by GO annotation, we built a PCA biplot based on the 12 gene expression profiles (Figure 2). We found that 12 DEGs assigned as “transcription activators (Transcription Factors, TF)” underlined by the particular GO term were quite antagonistically distributed (Figure 2). Note 3 species clusters therein: control (green shaded), AD (*Zic1, Zic2, Tcf7l2*), and A20/AD (*Zeb2, Foxi2, Pou2f2*) clusters.

**Figure 3:**
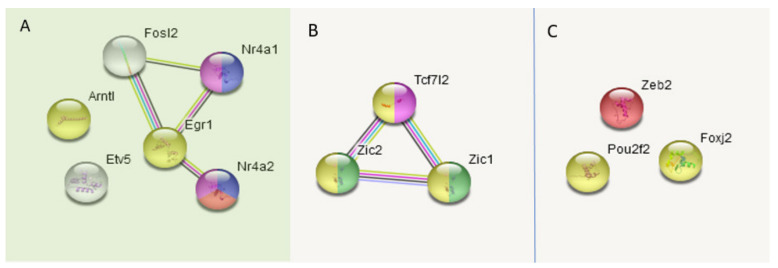
GO annotation of 12 TFs split into three corresponding clusters (**A**–**C**) (Figure 2). Overall PPI enrichment *p*-value (7 edges vs. 1 exp): 5.23 × 10^−6^. Green shaded area (**A**) is a control-group-associated cluster (Figure 2). Color coding: yellow—KW-0010: activator, 7 genes; FDR < 2.4 × 10^−6^; red—central nervous system projection neuron axonogenesis; 2 genes; FDR < 0.03; pink—GO:0035257; nuclear hormone receptor binding; 3 genes; FDR < 0.015; blue—GO:0071376 cellular response to corticotropin-releasing hormone stimulus; 2 genes; FDR < 0.0024; green—behavioral abnormalities of Zic1 and Zic2 mutant mice; PMID: 11699604. The image was created with the string-db.org service.

Consequent GO analysis underlined connected networks in two of three clusters, as depicted in Figure 3. 

In total, 145 publications referencing this particular 12 fold TF activator set are available, according to the “reference publications” term in the string-db.org resource. Three GO categories are valid throughout the whole 12 fold set: (1) DNA-binding transcription activator activity, RNA polymerase II-specific (12 genes; FDR < 4 × 10^−17^); (2) sequence-specific DNA binding (12 genes; FDR < 4.4 × 10^−13^); (3) nucleoplasm (12 genes; FDR < 2.7 × 10^−7^).

#### 3.2.1. Expansion of TF Clusters by Co-Variation Analysis

To gain further insight into the 12 TF features, we employed expansion of the DEG networks presented in Figure 3 by co-variation analysis of all gene profiles across 18-fold samples, using as the seeds the genes from the target 12 TF list (Figure 3). Expression profiles were assessed pairwise with the Kendall rank correlation coefficient.

We applied the algorithm of min/max optimization, aimed at elucidating the minimal connected neighborhood with the maximum target genes. For this, we used the genes with the highest correlation values with queries from the target list of six, three and three TF clusters (Figure 3) with a threshold of Kendall correlation coefficient: *r >* 0.6 (*p* < 1 × 10^−3^). 

#### 3.2.2. Control Six-TF DEGs Assessment

**Figure 4:**
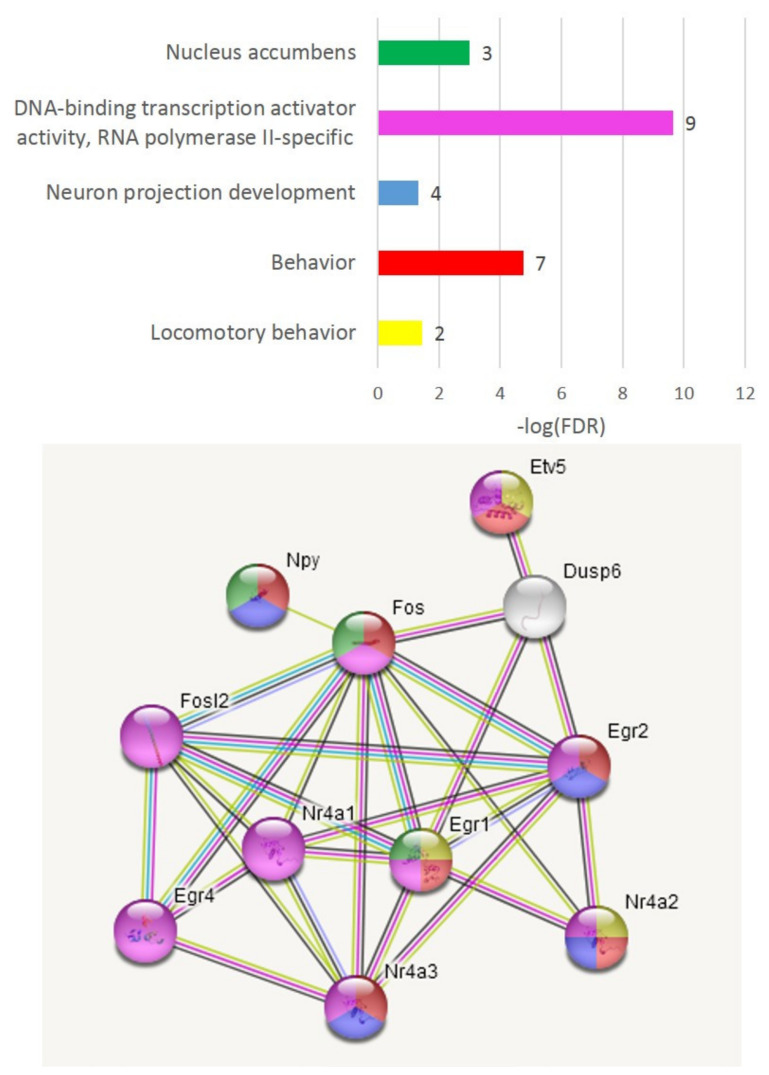
Eleven connected co-varied genes spanning 5 DEGs (*Egr1*, *Etv5*, *Fosl2*, *Nr4a1*, *Nr4a2*) presented in Figure 3A. GO color coding corresponds to the plot above (gene numbers attached to bars). The DEGs in this set feature control group and are decreased in AD and A20 groups (Figure 2).

As a result of the min/max algorithm, we efficiently expanded the “control” six-TF DEG cluster (Figure 3A) and generated a connected subset (11 genes) spanning five DEGs, as shown in Figure 4. We allowed the algorithm to include the genes from the same gene families even if this did not lead to the inclusion of the DEGs. In this way, we, as a rule, maintained a more robust gene network due to their regular relevance.

The majority were transcription factors related to the “regulation of neuronal synaptic plasticity” and “nervous system development” GO terms. Seven TFs were reported to affect behavior (Figure 4). All genes significantly correlated with the *Fos* TF, implying its priority in the set. The set comprising *Fosl2, Egr1, Etv5, Nr4a1* and *Nr4a2* was related to the cellular response to corticotropin-releasing hormone stimulus, cellular response to oxidative stress, locomotory behavior and response to hypoxia according to GO annotation (Figure 4).

#### 3.2.3. Circadian Rhythm DEG Analysis

**Figure 5:**
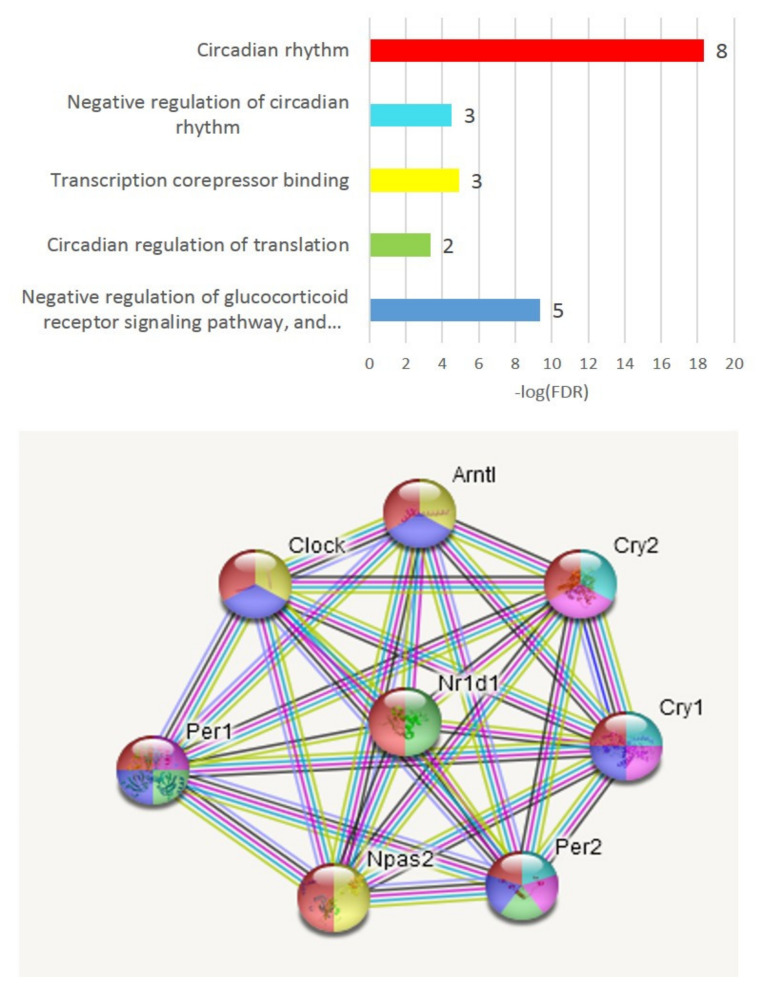
Eight circadian rhythm genes subset expanded from *Arntl* DEG and annotated by string-db suite (Figure 3A).

It appeared that the *Arntl* DEG (Figure 3A) was not related to other DEGs from the control group and was implicated in the circadian rhythm gene network (Figure 5).

**Figure 6:**
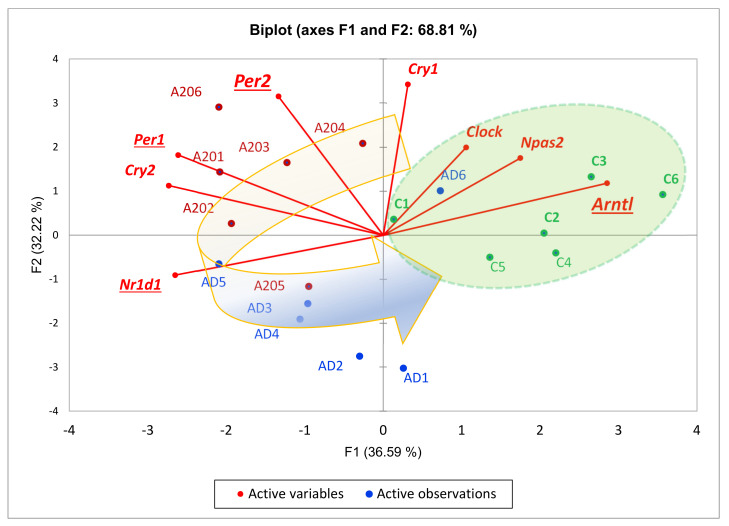
Circadian rhythm TF set (8 genes; Figure 5) PCA biplot featuring a control-group-associated *Arntl* DEG (Figure 3A) along with experimentally confirmed (string-db) connected genes *Npas2, Clock* (green shaded area) that are attenuated in affected species due to repressive action of cAMP-associated *Per1* expression in aggressive mice. *H0:* random distribution of species across plot’s 2 halves: *p* < 0.0024; binomial test. Each gene statistically significantly correlates with at least 2 other genes (*p* < 0.05) except for *Clock* (Table 2). Curved arrow depicts group-related sequential gene expression dynamics cycle: C->A20->AD->C. Underlined gene names indicate DEGs in any of the 3-way comparisons. Enlarged underlined genes feature differential expression in 2 comparisons: *Arntl* (C_AD, C_A20)*; Per2* (AD_A20, C_A20).

We underlined three repressive DEGs (*Per1*, *Per2*, *Nr1d1*) besides *Arntl* inferred from C_AD, AD_A20, C_A20 comparisons when we built a PCA plot across all mice groups based on eight gene expression profiles (Figure 6). Aggression-related genes repressive to circadian rhythm genes *Cry1*, *Cry2*, *Per1*, *Per2* were downturned in AD species, except for *Nr1d1*, but the key circadian genes (*Arntl*, *Clock*, *Npas2*) had not yet recovered their expression, implying an acute transition state in the AD group, featuring specifically high expression of the antagonistic *Nr1d1* DEG in the AD group (Figure 6). Notably, certain circadian clock genes negatively regulate the glucocorticoid receptor pathway (GO plot, top, Figure 5); thus, hormonal misbalance may take place given the shifted state of the gene expression profile dynamics (Figure 6).

The disruption of the circadian clock apparently followed increased dopamine uptake in aggressive mice, as underlined in [29]. 

#### 3.2.4. Cluster Two Expansion

**Figure 7:**
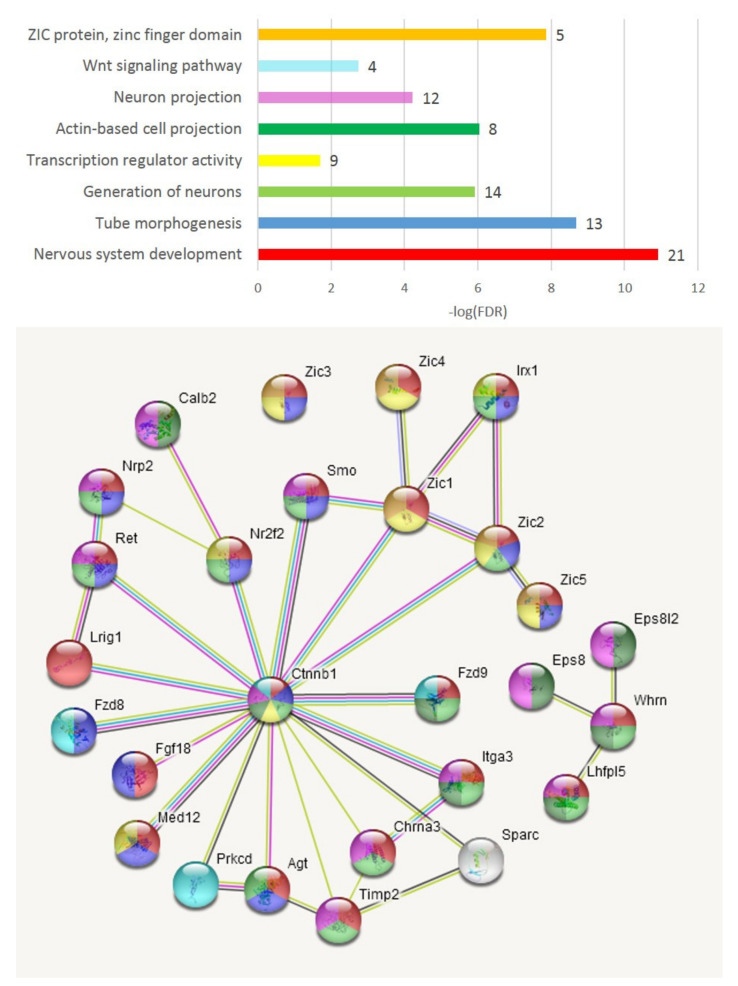
Twenty-seven genes recovered by string-db.org as Zic1/2-associated TF network and associated with AD subgroup (Figure 2 and Figure 3B). *Armadillo* (*Ctnnb1)* gene (catenin β 1) is the most connected gene in the center. GO color coding is assigned as in histogram plot above (number of genes is attached as bar labels therein).

**Figure 8:**
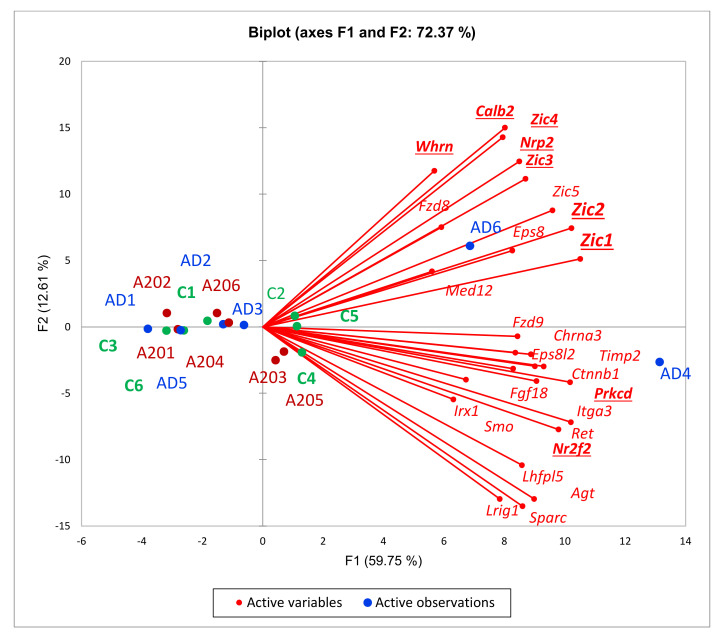
PCA plot of 27 genes from Figure 7 gene network of AD-specific *Wnt*-associated (*Ctnnb1*, *Prkcd*, *Fzd8*, *Fzd9*; [31]) TF program; see also Figure 2 and Figure 3. Bold underlined gene names indicate 9 DEGs within this network.

We assessed the expanded three-DEG set (Figure 3B) featuring the *wnt* signaling and consequent attenuation of neural development (Figure 7). The *Zic* family of zinc finger C2H2 proteins is annotated as transcriptional repressors (*Zic1-Zic5*). *Zic1* is reported to repress the *Drd1* receptor, characteristic of AD species lacking dopamine. The *Zic1*, *Zic2* genes are reported to be involved in behavioral abnormalities [30]. Overall, this cluster comprises several TFs (*Smo*, *Ctnnb1*, *Nr2f2*, *Zic2*, *Med12*, *Zic3*) involved in sustaining pluripotency (GO: WP1763; Figure 7), alongside transcription/development regulation, as well as overlapping with carcinogenic marker genes (*Ctnnb1*, *Fzd8*, *Fzd9*) in various tissues except for brain-related ones, as reported elsewhere. Figure 8 confirms these genes’ concordant expression in the AD mice subgroup. Notably, we recovered six more DEGs (*Calb2*, *Zic4*, *Prkcd*, *Nrp2*, *Nr2f2*, *Whrn*; Figure 8) present in the connected network (Figure 7), specifically in the A20_AD comparison, and the significance rate for all nine DEGs was the highest possible (Table S2). This gene cluster was downturned in A20 relative to the controls (see *Whrn* DEG twofold difference; Table S2). Overall, the C_AD comparison yielded four associated DEGs (*Prkcd*, *Calb2*, *Zic1*, *Zic2*) augmented specifically due to AD4 and AD6 species (Figure 2; Table S2). Based on this fact, we may state that the emergence of this particular cluster/network for AD4 and AD6 species (Figure 2) fundamentally antagonizes/contradicts A20’s gene expression pattern (as well as that of the control group; Figure 2 and Figure 8), and may be the basic cause of acute phenotype manifestation. *Whrn* DEG is associated with actin-based projection dynamics along with other genes (Figure 7), implying that membrane structure remodeling may be employed during the process.

The *Wnt* pathway essentially relates to the embryonic development program, implying high dynamics of transcription program evolvement in mature tissues as well [31]. It starts with Wnt* gene(s) transcription, which causes an accumulation of Catenin β (*Armadilio)* in the cytoplasm and its translocation to the nucleus, acting as a transcriptional coactivator of transcription factors. Thus, overexpression of *Ctnnb1* points to the exemplification of the *Wnt* program in the cells. 

Also, in regard to this path, we observed *Wnt9a, Wint10a* as DEGs elevated in AD mice, as featured in the AD_A20 comparisons with high confidence (FDR < 0.0014 for *Wnt9a*; Table S2). The C_A20 comparison manifests four *Wnt* DEGs (Table S2), including the abovementioned two in AD being highly depressed in A20, implying a transition activation mode of these genes in AD evolving from A20 to control group.

While *Tcf7l2 (*Figure 3B) is not physically associated with the AD-related DEG cluster depicted in Figure 7 and Figure 8, *Tcf7l2* manifests the broadest significant co-variation gene volume among the 12 DEGs considered. Its primary known functional partner is *wnt3.* The pair feature a carcinogenic tandem, as reported in many studies. Mostly glial genes’ expression profiles co-vary with *Tcf7l2*. After thorough analysis, we may state that it represents gene clusters involved in the *wnt* signaling response related to gliogenesis and myelination [31].

#### 3.2.5. Third Cluster Featuring Joint A20/AD Species

**Figure 9:**
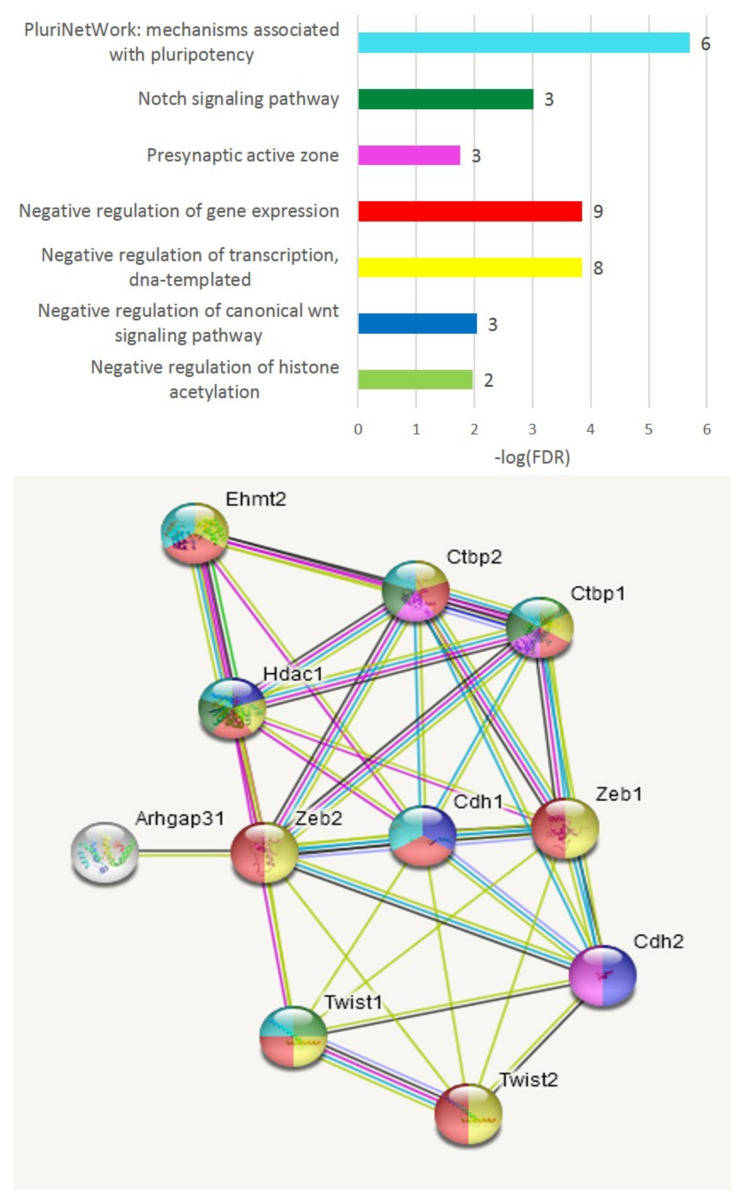
Neighborhood recovery of *Zeb2* neighborhood inferred by string-db.org resource (11 genes) implies “repression of transcription” GO function of genes set. All but one (*Arhgap*) genes are transcriptional repressors; 8 genes belong to nucleus compartment (TFs); 4 genes (*Cdh1*, *Cdh2*, *Ctbp1*, *Ctbp2*) are associated with glutamatergic synapse.

The three genes of A20-associated clusters are *Zeb2, Pou2f2* and *Foxj2.* The latter two are homeobox TF genes, while *Zeb2* is essentially a member of the effective transcriptional repression TF gene network related to sustaining a pluripotent state (Figure 9).

Thus, we may report that both affected species groups’ associated DEG TF clusters (Figure 3B,C) feature transcription repression gene networks accompanied by the consequent attenuation of positive development TFs featured in the control group (Figure 3A).

### 3.3. A20 vs. AD Comparison

We observed 62 DEGs in the C_AD comparison, while more than 1000 DEGs were featured in the A20 transcriptome against two others (Table 1; Table S2). Analysis of the C_AD DEGs comparison above implies that, except for 12 quite distinct and coordinated master TF gene alterations elucidated above, there are no other alterations between the C and AD groups subject to sensible results. Thus, we decided to limit our further analysis to the A20_AD comparison only, not considering the C_A20 one, since DEG overlap was quite dominant between C_A20 and AD_A20 (around 1000 DEGs). When appropriate, we used three-way comparisons in further PCA plots featuring the group specifics in each particular gene network.

#### 3.3.1. Differentially Expressed Genes between Groups

**Figure 10:**
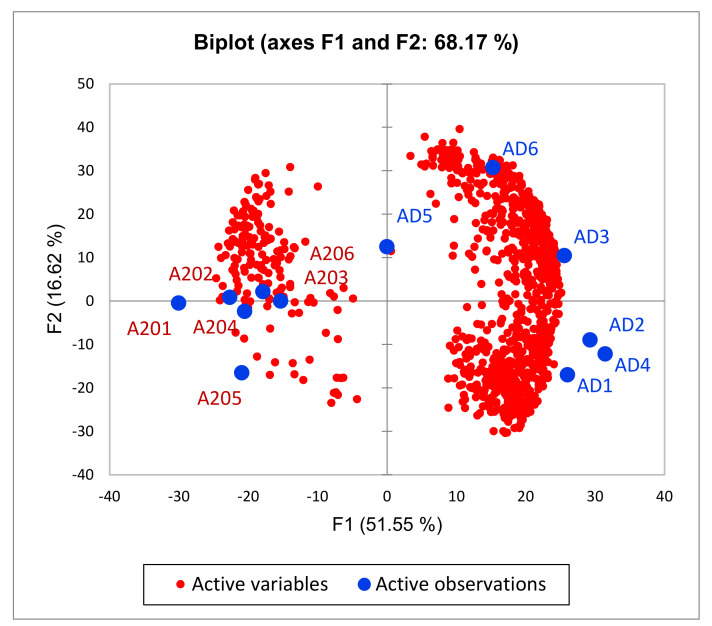
Total of 1009 DEGs across AD and A20 groups. A20 group maintained around 170 increased DEGs, while 839 DEGs featured attenuated expression in the A20 DS.

We started with outlining that the A20 group attenuated its transcriptome in a major part against the AD one, featuring 839 DEGs that decreased their expression, against 170 with increased expression profiles (Figure 10).

An overall breakdown of the GO categories in A20_vs_AD is presented in Table S3 in the graphical plots therein. In particular, there are 67 relevant pathways from KEGG, including cAMP-mediated signaling, learning and glutamate receptor pathways (Table S3). A total of 239 genes were involved in the annotated KEGG pathways from the DEG gene list. The majority of the KEGG pathways (45 from 67) partially included the cAMP signaling network. Both cellular component and biological process GO categories were highly enriched (high FDR significance) with neuron-specific compartments/pathways (Table S3). At least half of the DEGs (536) were phosphoproteins, implying regulation complexity and the signal transduction type in medium spiny as well as other neurons. Tissue type GO annotation unambiguously assigned most of its categories to brain tissues, including relevant corpus striatum/neostriatum/striatal neuron types (Table S3). A total of 18 “negative regulation” GO terms were observed in the “Biological Process” GO category (Table S3).

The primary analysis performed by clustering DEG by gene family (Table 3) revealed a great deal of genes involved in synaptic plasticity, as previously reported. In particular, we observed 8 Rho GTPAses (Arh), 10 collagens, the Kif family (6DEGs), regulators of G protein signaling (5 DEGs), etc. (Table 3). From 192 DEGs in the families with DEGs > 4 in Table 3, only 24 DEGs increased their expression in the A20 group.

#### 3.3.2. Elaborating on A20-Associated Positive DEG Cluster

**Figure 11:**
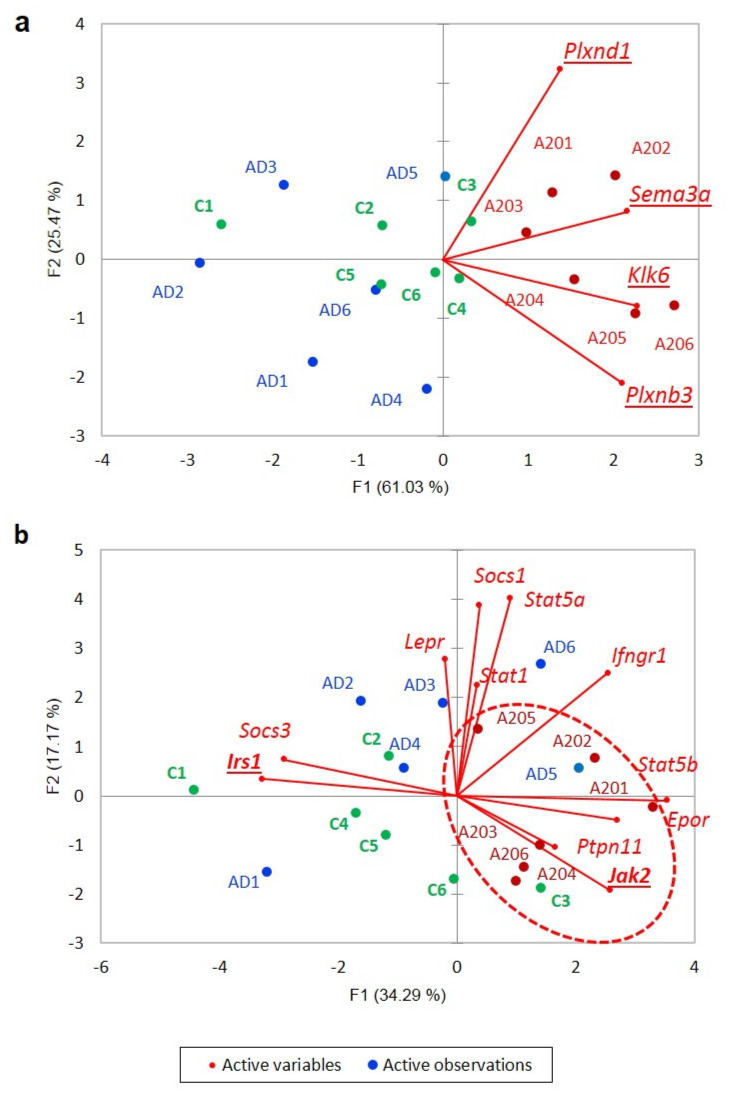
(**a**) “Semaphorin-plexin signaling” GO DEG set (4 genes); *H0:* random distribution of samples across 2 plot halves, *p*-value < 0.00065 (binomial test); (**b**) JAK/STAT gene projection inferred from string-db.org seeded by *Jak2, Irs1* DEGs; *H0:* random distribution of samples across 2 plot halves, *p*-value < 0.0045 (binomial test). Underlined gene names signify DEGs. Encircled is A20 group.

We selected 170 DEGs elevated in the A20 group (Figure 10) to assess the functional specifics of the DEGs located within. Upon the clustering routine, we observed virtually a single cluster of 149 DEGs manifesting the cAMP-dependent signaling cascade and associated DEGs, while 21 other DEGs presented non-specific singletons/small clusters. According to GO annotation, we observed a large set of “negative regulation of cellular process” GO enrichment DEGs (58 genes; FDR = 2.3 × 10^−4^). The “semaphorin-plexin signaling pathway involved in axon guidance” repression pathway (*Sema3a, Pxnd1, Klk6, Plxnb3* DEGs; FDR < 0.0146) was featured in the A20 group (Figure 11a). The dopamine-mediated JAK/STAT pathway gene network reported to be evoked in Parkinson disease [32] also proved to be elevated specifically in the A20 group (*Jak22, Irs1* DEGs; Figure 11b). Explicit GO annotation of other GO-enriched categories of the positively regulated 149 DEG cluster in the A20 group is available in Table S5.

Notably, the cAMP cluster conveyed some facts that are worth noting. First, synaptic scaffold genes *PSD95 (Dlg4)* and *Homer1* increased their expression in the DS of aggressive species due to the impact of the cAMP-mediated cascade (and, hence, opioid exposure), contrary to what was reported in [18] for the NAcc region. We should state that *Dlg4* performs a specific role in glutamate/dopamine receptor switching in MSNs, as reported in [33,34,35]. *Homer1* was reported to be increased in the amygdala of opioid users [36]. It should be stated, though, that the increase was not prominent (not significant in *Dlg4* and minimally significant in *Homer1* (FDR=0.041; see Table S3)). No significantly different expression of these genes in the C_A20 comparison was observed since the control group featured more active dopamine uptake in the DS (Table S3). Nonetheless, one should bear in mind that we dealt with a relatively mild endogenous opioid synthesis impact in the A20 group.

**Circadian entrainment genes** were among the non-randomly enriched DEGs in the A20_AD comparison associated with the cAMP cascade as well (mmu04713; *Gucy1b3, Rps6ka5, Gucy1a3, Per2, Gnb5, Cacna1h*; FDR < 0.024). The “negatively regulated circadian rhythm” GO enrichment contained DEGs (*Cry1, Drd1, Per2, Drd2*), implying a connection of circadian clock genes *Cry1, Per2* with the cAMP-dependent cascade. Indeed, we observed the repression of positive circadian rhythm genes’ expression *Npas2, Arntl* by *Cry1, Per2* in A20 and AD species (Figure 6).

**Figure 12:**
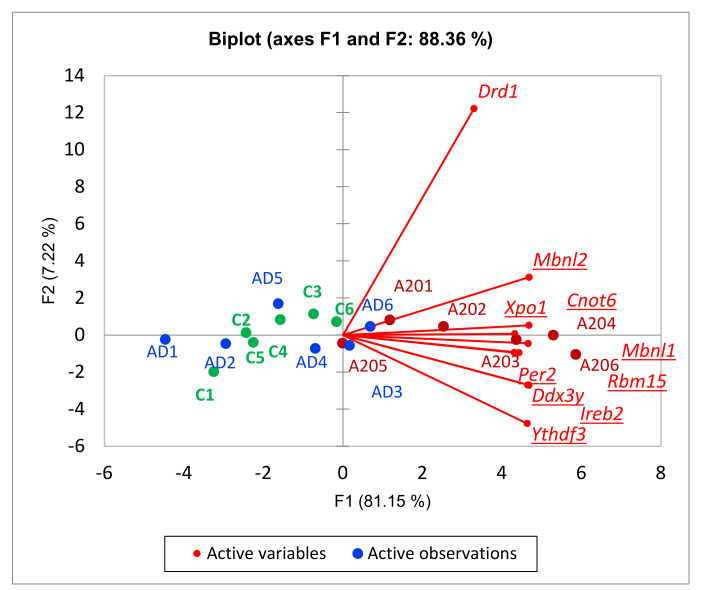
RNA-binding DEG subcluster within 149 cAMP-mediated positive A20 DEG cluster (Figure 10). *Drd1* included, underlining significant correlation with these DEGs. *Per2, Rbm15* are known transcriptional repressors (see the text). *H0:* random distribution of samples across 2 plot halves, *p*-value < 0.0045 (binomial test). Underlined gene names signify DEGs.

Finally, we report a subcluster of highly correlated RNA-binding proteins (*Cnot6, Ddx3y, Ireb2, Mbnl1, Mbnl2, Per2, Rbm15, Xpo1, Ythdf3*), depicted in Figure 12. Three of them (*Mbnl1, Mbnl2, Rbm15)* represent splicing factors. Overall, the cluster manifests negative regulation of transcription. In particular, *Rbm15* is reported to facilitate m6a posttranscriptional methylation of mRNA [37]. To the best of our knowledge, we are the first to report such DEGs in regard to DS cAMP signaling.

#### 3.3.3. Dopamine-Mediated cAMP Signaling Cascade Balanced Gene Projection in A20 vs. AD Comparison

**Figure 13:**
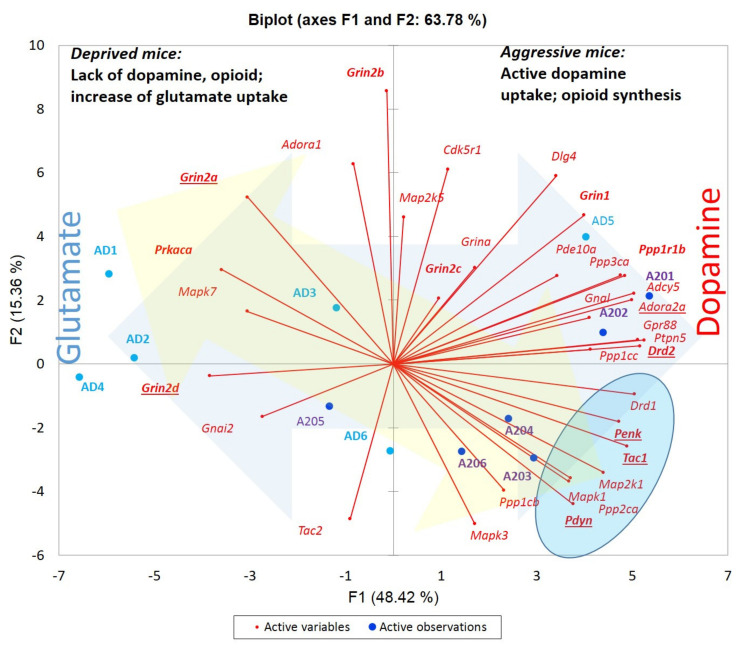
PCA plot based on 33 specific cAMP-mediated balanced gene subset compiled in (Babenko et al., 2020) featuring A20 and AD groups. Marker gene set is shown in bold. Blue-shaded arrow signifies dopamine vs. glutamate uptake gradient. Yellow-shaded arrow signifies D1 passive/active states phasing [11]. Blue-shaded oval underlines opioid synthesis (*Penk, Pdyn). H0:* random distribution of 5-fold AD sample with only 1 alien species in a plot; left half is *p*-value < 0.016 (binomial distribution). Blue dots are AD samples; dark blue dots are aggressive samples. Underlined gene names indicate DEGs.

We underlined two basic interconnected gene groups relevant to the aggression manifestation we consider keystone according to previous publications on behavioral genetics [7], which are the cAMP-mediated gene network and associated endogenous opioid expression. We assessed samples based on the cAMP-mediated dopamine turnover balanced genes set comprising 33 genes introduced in our previous work [11]. The PCA plot underlines high MSN dopamine uptake accompanied by endogenous opioid synthesis in aggressive species (Figure 13). Oppositely, based on *Drd1, Drd2* DEGs and *Ppp1r1b (Darpp-32)* location we infer profound dopamine uptake depression in AD species, along with the abrogation of opioid synthesis.

## 4. Discussion

We report that the majority of DEGs in the AD_A20 comparison are attributed to synaptic remodeling (GO terms: neuron development, etc.; Table S3) and the attenuation of major development/metabolic processes, leading to the overall downregulation of 839 DEGs from 1009 (Figure 10).

Analysis of DEGs cluster (170 entries) with increased expression in aggressive mice outlined the major role of the cAMP-mediated response in the dopamine uptake gene network and several associated ones, including multiple repressive TFs networks.

**Figure 14:**
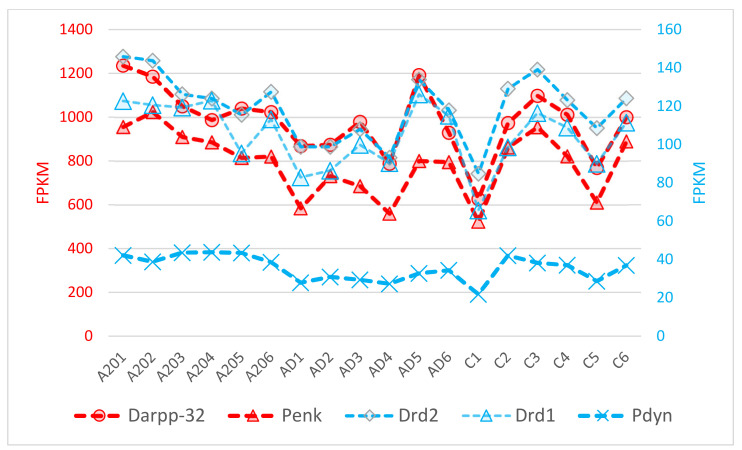
FPKM values of 5 dopamine- and opioid-specific genes across 18 samples.

To gain further insight into the dopamine–opioid interactions, we inferred that the most expressed striatum neuron-specific genes observed in our samples were *Darpp-32* (avg. exp. 1000 FPKM) and *Penk* (800 FPKM) reported elsewhere. We added dopamine-specific gene *Drd1/2* and opioid-associated *Penk* and *Pdyn* genes for comparison, as presented in Figure 14. Both *Darpp-32* (*Ppp1r1b)* and *Penk* are the highest expressed DS coding neuron specific genes attributed to MSN.

We utilized the balanced dopamine cAMP mediated signaling gene network depicted in Figure 13 elucidating an expression projection of this cascade implicitly reflecting the signaling intensity specifics. It is worth noting that incorporated *Darpp-32*, the ‘heart’ of dopamine mediated signaling cascade assures high confidence of elevated dopamine uptake by its expression shift (Figure 13) supported by coordinated *Drd1, 2* DEGs expression. At the same time, it’s hard to ever expect it being DEG due to a high variation (Figure 14) and given enormous expression rate. 

One of the basic observations supporting the validity of our analysis is the observed high co-variation of striatum-specific dopamine and opioid genes, outlined in Table 4 and based on data from Figure 14. We found the five genes considered co-vary non-randomly with *p*-value < 1 × 10^−7^. *Pdyn* is less coordinated (*p* < 1 × 10^−5^; Table 4), less expressed than others (30 FPKM avg.; Figure 14) and is not strictly striatum-specific (hypothalamus, some other regions). Nonetheless, due to its rather stable expression mode it is the most significant DEG among the cAMP set, along with *Drd2* (DEG significance for both: FDR < 0.00135, the maximum possible; Table S2).

The reason for such a high correlation rate is that all genes considered are subject to cAMP-mediated activation, including *Penk* [38]. We thus reconfirm the inherent opioid synthesis upon dopamine uptake reported elsewhere [13,14], and, based on our analysis, we considered these coordinated networks to be the most causal ones.

After elucidation of the core DEG networks, we conclude that high dopamine uptake accompanied by endogenous opioid synthesis in A20 results in profound alteration of the dorsal striatum transcriptome, leading to more than 1000 DEGs in A20 compared to other groups, and more than 540 (though highly overlapping) GO processes’ enrichment (Table S3). These changes feature many gene circuits/processes outlined for AD_A20 GO annotation (Table S3). We specifically underline dopamine-mediated cAMP signaling featuring an increase in the opioidergic network (Figure 13; blue oval). From 63 KEGG networks’ enrichment, 45 overlap with the cAMP cascade (Table S3; KEGG spreadsheet).

Two weeks of aggressive species fight deprivation led to significant restoration of the normal transcriptome in AD (Table 1). A lack of opioid and dopamine uptake in the AD dorsal striatum followed profound GABA reuptake, indicated by a high *Slc6a11* expression increase in AD vs. A20 (*q* < 0.00135; Table S3), implying that MSN GABAergic signaling stalls in AD species. 

Previous studies underline the profound alteration of opioid-induced synaptic plasticity in MSNs, including structural, transcriptional and epigenetically invoked components [21]. We report distinct structural synaptic remodeling dynamics, observing “actin filament-based movement”, “actin-mediated cell contraction”, “actin filament-based process” (50 DEGs) and “regulation of actin filament-based process” (23 DEGs) GO enrichment significantly shifted in A20 from the AD/control. In particular, we report 41 DEGs of “actin cytoskeleton organization” enrichment (Table S3; “Process” sheet; s.669; FDR < 0.007; GO:0030036), corresponding with the earlier connection with the synaptic membrane [39]. Virtually all of the DEG networks mentioned in the GO enrichment (Table S3) were downregulated in the A20 group. 

Regarding C_AD DEGs, we report three clusters of TF activators (Figure 3), two featuring repressors augmented in A20/AD species, with a corresponding attenuation of the control TF activators DEG cluster. It is worth noting that we elucidate circadian rhythm network disruption in all three transcriptome comparisons, which was significant in both the aggressive and aggression-deprived groups (Figure 6). Previous studies underlined the withdrawal effect of the *Per2* gene [40], as well as its dopamine-associated expression pattern [41], featuring abrogation of the withdrawal effect while knocking *Per2* out [42]. Notably, we observed a significant downturn in *Per2* in AD mice (Figure 6), implying a transition to a normal state. We point out the *Nr1d1* DEG as the most antagonistic one in the AD group relative to the control group within the circadian clock gene network (Figure 6). Overall, three repressive DEGs (*Per1, Per2, Nr1d1)* manifest the greatest discrepancy with control group circadian genes in affected species, as reported in [43]. The effects on circadian clock genes upon chronic opioid exposure were also reported in a series of recent publications [44,45,46].

**Figure 15:**
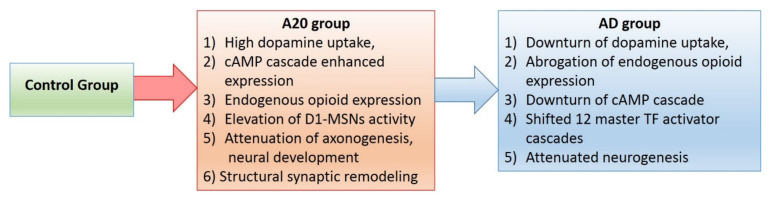
Flowchart of DEG inferred events from aggressive to control groups.

We performed RNA-Seq analysis of aggressive (A20), aggression-deprived (AD) and control groups, evaluating their relationships. We present our major results in the relation diagram in Figure 15, elucidated by DEG analysis. Therein, the red arrow underlines a high expression discrepancy (more than 1000 DEGs; Table 1). The blue arrow indicates quite few DEGs (given C_AD comparison; Table 1), but they were quite empowered TF activators (Figure 3), producing a non-significant distribution but a considerable effect on the key neurogenetic networks related to withdrawal syndrome after opioid addiction. Notably, a downturn in opioid synthesis in aggressive mice was reported in [47], but the authors examined the whole brain tissue. The current study links dopamine-mediated increased opioid expression with the DS in aggressive mice (Figure 13 and Figure 14), as also confirmed in our previous studies [2,3,4].

## 5. Conclusions

We report high overall attenuation of the DS transcriptome expression rate (170 out of 1009 DEGs are upregulated in A20) upon aggression-related stress, featuring high dopamine uptake and endogenous opioid synthesis. Strikingly, we observed quite rapid (14-day period) recovery of the major body of transcriptome gene expression profiles upon fighting round abrogation, with the unique elevation of 12 TF activators’ expression profiles. Most of these DEG TFs refer to the repression of neuron development, which, in particular, implies synaptic scaffold rearrangement. We assume, given the contraction of the postsynaptic density architecture [18,19], along with the alteration of synaptic gene expression upon opioid exposure, that it takes considerable time to restore the intact synapse plasticity transcription program along with the circadian rhythm, in this way leading to the withdrawal symptoms.

Concerning the A20 group, we report that the cAMP-mediated gene cascade displays significant elevation following the disruption of the circadian clock, increased opioid synthesis, the evocation of *Wnt* signaling in the DS and a downturn in overall neurogenesis and glial development. We underline the similarity of the DS and NAcc brain regions based on neuron content (MSNs as majority; [10]), thus manifesting similar mechanisms within this core, including maintaining dopaminergic projections from VTA and employing cAMP-mediated intracellular signal cascades within them.

As group-wise specific features, we report neuroinflammation related Semaphorin-plexin and JAK/STAT signaling instantiations in A20 group (Figure 11), and wnt cascade being robustly manifested in part of AD group (Figure 8), possibly linked to synaptic plasticity restoration including dendritic spines and axon projections architecture lost upon endogenous opioid exposure [21].

## Figures and Tables

**Table 1:** Three-way comparisons outline based on *CuffDiff* [26] DEG detection.

	Number of DEGs (FDR < 0.05)	Number of Non-Zero Expressed Gene Pairs
**Control vs. A20**	1030	24,321
**Control vs. AD**	62	23,924
**A20 vs. AD**	1009	23,763

**Table 2:** Pearson pairwise correlation matrix; df = 17.

Variables	*Nr1d1*	*Cry2*	*Per1*	*Npas2*	*Arntl*	*Cry1*	*Clock*	*Per2*
*Nr1d1*	**1**	**0.603**	0.312	−0.247	**−0.620**	−0.288	−0.305	0.025
*Cry2*	**0.603**	**1**	**0.656**	−0.087	−0.392	0.179	−0.138	0.447
*Per1*	0.312	**0.656**	**1**	−0.226	**−0.474**	0.297	0.126	**0.669**
*Npas2*	−0.247	−0.087	−0.226	**1**	**0.552**	**0.510**	0.223	0.065
*Arntl*	**−0.620**	−0.392	**−0.474**	**0.552**	**1**	0.325	0.369	−0.067
*Cry1*	−0.288	0.179	0.297	**0.510**	0.325	**1**	0.323	**0.786**
*Clock*	−0.305	−0.138	0.126	0.223	0.369	0.323	**1**	0.274
*Per2*	0.025	0.447	**0.669**	0.065	–0.067	**0.786**	0.274	**1**

Values in bold are different from 0 with a significance level α = 0.05.

**Table 3:** Abbreviated gene families and #DEGs per family. The majority represent synaptic-associated genes and ion transporters (*Slc*). (See Table S4 for expanded info.) *q*_value = 1.35 × 10^−3^ is the minimal value throughout the transcriptome of 23 thousand genes signifying the maximal accuracy threshold (virtually zero) defined by a multiple-comparisons correction value.

Family	Min (*q*_Value)	Max (*q*_Value)	Stddev (*q*_Value)	# DEGs per Family
Slc	1.35 × 10^−3^	3.63 × 10^−2^	1.05 × 10^−2^	29
Kcn	1.35 × 10^−3^	4.92 × 10^−2^	1.31 × 10^−2^	22
Tme	1.35 × 10^−3^	4.88 × 10^−2^	1.50 × 10^−2^	17
Zfp	1.35 × 10^−3^	4.97 × 10^−2^	1.38 × 10^−2^	15
Col	1.35 × 10^−3^	4.39 × 10^−2^	1.28 × 10^−2^	10
Arh	1.35 × 10^−3^	4.88 × 10^−2^	1.66 × 10^−2^	8
Ptp	1.35 × 10^−3^	2.59 × 10^−2^	1.04 × 10^−2^	7
Map	1.35 × 10^−3^	2.31 × 10^−2^	7.40 × 10^−3^	7
Gpr	1.35 × 10^−3^	2.10 × 10^−2^	7.21 × 10^−3^	7
Kif	1.35 × 10^−3^	2.24 × 10^−2^	7.86 × 10^−3^	6
Cac	1.35 × 10^−3^	4.56 × 10^−2^	1.89 × 10^−2^	6
Tri	1.35 × 10^−3^	3.10 × 10^−2^	1.30 × 10^−2^	6
Ank	1.35 × 10^−3^	4.56 × 10^−2^	1.51 × 10^−2^	6
Fam	1.35 × 10^−3^	1.37 × 10^−2^	4.58 × 10^−3^	6
Cdk	1.35 × 10^−3^	4.45 × 10^−2^	1.72 × 10^−2^	5
Rgs	1.35 × 10^−3^	4.67 × 10^−2^	1.57 × 10^−2^	5
Prr	1.35 × 10^−3^	3.76 × 10^−2^	1.38 × 10^−2^	5
Aka	1.35 × 10^−3^	2.51 × 10^−2^	9.33 × 10^−3^	5
Ple	1.35 × 10^−3^	4.78 × 10^−2^	1.94 × 10^−2^	5
Cep	1.35 × 10^−3^	4.63 × 10^−3^	1.28 × 10^−3^	5
Adc	1.35 × 10^−3^	4.63 × 10^−3^	1.39 × 10^−3^	5
Doc	1.35 × 10^−3^	9.39 × 10^−3^	3.13 × 10^−3^	5

**Table 4:** Pearson pairwise correlation coefficients of key DS-specific genes’ expression profiles across samples (df = 17).

Variables	*Darpp-32*	*Penk*	*Drd2*	*Drd1*	*Pdyn*
*Darpp-32*	**1**	**0.858**	**0.914**	**0.863**	0.723
*Penk*	**0.858**	**1**	**0.925**	**0.838**	**0.867**
*Drd2*	**0.914**	**0.925**	**1**	**0.886**	0.777
*Drd1*	**0.863**	**0.838**	**0.886**	**1**	0.717
*Pdyn*	0.723	**0.867**	0.777	0.717	**1**

Values in bold are different from 0 with a significance level α = 1 × 10^−7^.

## Data Availability

The RNA-Seq datasets are available in the European Nucleotide Archive (Accession No. PRJEB48789).

## Supplementary Materials

The following are available online at https://www.mdpi.com/article/10.3390/genes13010021/s1, Table S1. Stats on mapping routine.xlsx, Table S2. DEGs table.xlsx, Table S3. GO AD_A20 DEGs comparison.xlsx, Table S4. Enriched families in A20_AD DEGs.xlsx, Table S5. 149 Positively regulated genes GO annotation.

## Abbreviations

DEGs	differentially expressed genes
FPKM	fragments per kilobase of transcript per million mapped reads
GO	Gene Ontology
KEGG	Kyoto Encyclopedia of Genes and Genomes Pathway Database
